# Predicting Unscheduled Emergency Department Return Visits Among Older Adults: Population-Based Retrospective Study

**DOI:** 10.2196/22491

**Published:** 2021-07-28

**Authors:** Rai-Fu Chen, Kuei-Chen Cheng, Yu-Yin Lin, I-Chiu Chang, Cheng-Han Tsai

**Affiliations:** 1 Department of Information Management Chia-Nan University of Pharmacy and Science Tainan City Taiwan; 2 Department of Information Management National Chung Cheng University Chiayi County Taiwan; 3 Department of Emergency Chiayi Branch, Taichung Veterans General Hospital Chiayi City Taiwan

**Keywords:** classification model, decision tree, emergency department, older adult patients, unscheduled return visits

## Abstract

**Background:**

Unscheduled emergency department return visits (EDRVs) are key indicators for monitoring the quality of emergency medical care. A high return rate implies that the medical services provided by the emergency department (ED) failed to achieve the expected results of accurate diagnosis and effective treatment. Older adults are more susceptible to diseases and comorbidities than younger adults, and they exhibit unique and complex clinical characteristics that increase the difficulty of clinical diagnosis and treatment. Older adults also use more emergency medical resources than people in other age groups. Many studies have reviewed the causes of EDRVs among general ED patients; however, few have focused on older adults, although this is the age group with the highest rate of EDRVs.

**Objective:**

This aim of this study is to establish a model for predicting unscheduled EDRVs within a 72-hour period among patients aged 65 years and older. In addition, we aim to investigate the effects of the influencing factors on their unscheduled EDRVs.

**Methods:**

We used stratified and randomized data from Taiwan’s National Health Insurance Research Database and applied data mining techniques to construct a prediction model consisting of patient, disease, hospital, and physician characteristics. Records of ED visits by patients aged 65 years and older from 1996 to 2010 in the National Health Insurance Research Database were selected, and the final sample size was 49,252 records.

**Results:**

The decision tree of the prediction model achieved an acceptable overall accuracy of 76.80%. Economic status, chronic illness, and length of stay in the ED were the top three variables influencing unscheduled EDRVs. Those who stayed in the ED overnight or longer on their first visit were less likely to return. This study confirms the results of prior studies, which found that economically underprivileged older adults with chronic illness and comorbidities were more likely to return to the ED.

**Conclusions:**

Medical institutions can use our prediction model as a reference to improve medical management and clinical services by understanding the reasons for 72-hour unscheduled EDRVs in older adult patients. A possible solution is to create mechanisms that incorporate our prediction model and develop a support system with customized medical education for older patients and their family members before discharge. Meanwhile, a reasonably longer length of stay in the ED may help evaluate treatments and guide prognosis for older adult patients, and it may further reduce the rate of their unscheduled EDRVs.

## Introduction

### Background and Setting

Many countries today face challenges related to the rapidly aging population. Advances in medical technology and the aging of post–World War II baby boomers have led to a greater proportion of adults aged over 65 years in many industrialized nations’ populations. This substantive shift in demographics not only increases the overall demand for health care and medical services but also influences economic and social welfare policies. Older adults are more susceptible to diseases and comorbidities than younger adults, and they exhibit unique and complex clinical characteristics that increase the difficulty of clinical diagnosis and treatment [1]. Older adults also use more emergency medical resources than people in other age demographics do [2-8], and approximately 14.9% of emergency department (ED) patients in the United States are aged 65 years or older [9], making them the most frequent visitors to the ED. In Taiwan, 25.5% of all ED visits are made by adults aged 65 years or older [10], a percentage that is approximately two-fold higher than that in the United States. This age group has the highest rate of ED return visits (EDRVs) [11,12].

A high unscheduled EDRV rate implies that the medical services provided by the ED failed to achieve the expected results of accurate diagnosis and effective treatment [11] and is a key indicator for monitoring the quality of emergency medical care [11,13]. EDRVs might contribute to crowding and further diminish the quality of care in the emergency room. As most older individuals have complex clinical characteristics, EDRVs would use more emergency room resources. In addition, EDRVs increase the risk of contracting infectious diseases in older adults, especially during the COVID-19 pandemic.

Many studies have reviewed the causes of EDRVs among general ED patients [11,14-16]; however, few have focused on older adults, even though this is the age group with the highest rate of EDRVs [11,12]. In fact, the risk of EDRVs for adult ED patients aged older than 65 years is approximately 300% higher than that for adults aged less than 30 years and 200% higher than that for adults aged less than 46 years [11,12,17]. Older adults who repeatedly return to the ED to seek medical assistance are at an increased risk of medical errors and contribute to excessive use of emergency medical resources [11,18]. However, the findings of past studies that did not focus on this demographic may not be suitable for predicting older adults’ rate of unscheduled EDRVs. In addition, past studies have mainly collected samples from single targets (hospitals) [11,12,14-17]. Sample collection from a single hospital source can lead to underestimation of return rates, because ED patients who return within 72 hours may visit a different hospital’s ED.

Taiwan’s health care services have been ranked the highest worldwide by The Richest [19] and second ranking worldwide by the Economist Intelligence Unit [20]. Numbeo ranked the health care system of Taiwan’s national health insurance first worldwide in its Health Care Index and Health Care Exp Index [21], with an enrollment of approximately 99.68% of the population [22]. The high ranking of Taiwan’s Digital Government Program [23] made the population-wide database, the National Health Insurance Research Database (NHIRD). Therefore, this study uses the NHIRD and develops a simple and useful prediction model to identify critical factors influencing older adult patients’ unscheduled EDRVs through a machine learning technique. Such a model could provide useful suggestions for hospital managers and health care professionals in delivering high ED qualities from the considerations of disease, patient, physician, and institution (hospital) factors. Furthermore, it could serve as a valuable reference for future government planning and promotion of medical services and age-friendly policies.

### Related Studies

The factors influencing EDRVs can be categorized into approximately four areas: disease-related, patient-related, physician-related, and medical institution–related factors. One of the major disease-related reasons for ED visits is a pathological condition with unclear symptoms, signs, and diagnoses, and the primary pathological condition responsible for EDRVs, such as abdominal pain [12,15-17,24-26] with a diagnostic error rate of 68%-73% [15]. Fever is another pathological condition that causes EDRVs [24,27]. Other disease-related factors include infectious disease [25] with urinary tract infections, accounting for 35% of all infectious diseases [12]; muscle, bone, or head traumas [14,26]; cancer [12,27]; and alcoholism, depression, and other mental illnesses. Patients with high triage classification (TC) [24,25,28], heart disease or diabetes [16], or chronic illness with comorbidities [17] also exhibit a high likelihood of an EDRV. A high Charlson Comorbidity Index indicates a high risk of EDRV [29,30], particularly for patients aged older than 75 years [25].

EDRVs are known to increase concurrently with age [11,12,17]. The gender effect on the rate of EDRVs is uncertain [16,24,26,31,32]. Other patient-related factors that have a significant influence on EDRVs include personal insistence on using ED services [33]. EDRVs are 25%-30% higher in low-income countries than in high-income countries [24]. Diagnostic errors by medical staff account for the highest percentage (5.7%-9%) of medical errors [27] and are a common cause of unscheduled EDRVs. Prior studies found that physicians’ years of practice significantly influenced the rates of EDRVs [15,34]. Kuan and Mahadevan [15] indicated that the reasons for physicians’ years of practice significantly influence the rates of EDRVs are related to their experience and training as ED physicians. Improved communication between physicians and staff, patients, and family members can also reduce the likelihood of EDRVs. Inadequate emergency resources, particularly in rural hospitals [31] or in staffing during nighttime and on weekends [13], increase the likelihood of EDRVs. An ED stay of more than 6 hours is uncommon [18] because longer stays might contribute to crowding problems and diminish the quality of providing expeditious triage, workup, and selection of endangered emergency patients.

In summary, the literature confirms that disease-, patient-, physician-, and institution-related factors all influence the rate of unscheduled EDRVs. As older patients’ EDRVs are associated with high risks and high impacts, this study focused on older patients and investigated the effects of the aforementioned influencing factors on their unscheduled EDRVs.

## Methods

### Research Procedures

The study was divided into two stages. The first stage entailed data selection and preprocessing. The second stage entailed data analysis. Machine learning techniques are unlikely to be restricted by statistical analysis assumptions or affected by collinear interactions between independent variables, and they demonstrate superior fault tolerance and learning capability. This study focuses on investigating the factors influencing the classification of 72-hour unscheduled EDRVs. The decision tree technique, one of machine learning classification techniques, is easier to interpret by a nonstatistician and is intuitive to follow compared with other methods (eg, random forest and support vector machine) [35,36]. In addition, decision trees have been widely used in various clinical studies for classification and prediction [37-41], and the analyzed results can be easily applied to clinical practice. Therefore, we used the decision tree as the major analysis method in this study. We used Weka (University of Waikato), one of the most popular machine learning tools, to perform in-depth data analysis for verification.

### Data Selection

We used the NHIRD as the data source and selected records of ED visits by patients aged 65 years or older from 1996 to 2010 and had older adult visits of 162,264 records out of 1,425,335 total ED visits. We then excluded 190 records of deaths and 26,912 records hospitalized within 72 hours after the ED visit. In 2010, Taiwan’s Ministry of Health and Welfare amended the emergency TC from four to five classes. To prevent data inconsistency, 21,318 records following the new emergency triage reclassification were excluded from the scope of this research. Meanwhile, the Ministry of Health and Welfare that launched improvements in medical technologies in 2005 might significantly influence the number of unscheduled EDRVs; therefore, 44,114 records from 1996 to 2004 were removed. Finally, 20,478 records with incomplete or illogical values were excluded to ensure the accuracy and consistency of the analyzed data. The final sample size was 49,252 records, including 3510 unscheduled EDRV records within 72 hours.

### Variables

To develop a prediction model for older patients’ unscheduled EDRVs, we applied the presence or absence of a *72-hour unscheduled EDRV* as the dependent variable. Patient-, disease-, hospital-, and physician-related characteristics were applied as independent variables. Patient-related characteristics included sex, age, economic status (ES), major disease or injury, and chronic illness (eg, hypertension, diabetes, heart disease, bowel dysfunction, cerebrovascular disease, chronic kidney inflammation, vestibular disease, mental illness, arthritis, and cancer drug treatment and monitoring). Disease-related characteristics included TC, diagnostic categories (DC), radiography examination (x-ray test, specific angiography, and ultrasound scan), surgery disposition, disease severity (DS), and length of stay in the ED (LOSED). Hospital-related characteristics comprised the level of hospital and level of urbanization (LU). Physician-related characteristics included gender, years of practice, and specialty.

Among the aforementioned independent variables, only age was a continuous variable; all other variables were categorical variables with a nominal or ordinal scale. Moreover, the variables of chronic illness and radiography examination comprised several subvariables. Detailed information related to the included variables is presented in Multimedia Appendix 1.

### Analyzed Method

We applied the C4.5 technique (ie, J48 in Weka) to create a decision tree for the classifications. Decision trees use a simple tree structure to represent a set of IF-THEN rules between independent and dependent variables. The tree structure consists of multiple internal and leaf nodes. In a decision tree, each internal node represents a single independent variable, each branch of a node represents one possible value or a set of possible values of the independent variable, and each leaf node represents a class label.

A 10-fold cross-validation method was used to randomly partition the data set into 10 subsets. The validation was repeated 10 times. A confusion matrix was established to evaluate the performance of the classification model. Subsequently, we calculated the average accuracy rate of the classification results for the 10 testing sets. The sensitivity and specificity were also examined. Sensitivity refers to the ability of the prediction model to accurately predict the EDRVs among the sampled population, whereas specificity refers to the ability of the prediction model to accurately predict the samples with no return to the ED; accuracy refers to the accuracy of the prediction model regardless of return or nonreturn to the ED.

The final sample size was 49,252 records, including 3510 unscheduled EDRV records within 72 hours. However, the number of unscheduled EDRVs within 72 hours indicated only 7.13% (3510/49,252) of the emergency visits (not unscheduled EDRVs). This raises a class imbalance problem, which may lead the rare class (unscheduled EDRVs) to be ignored in the prediction model. To overcome this problem, we maintained an approximately 1:1 ratio of unscheduled EDRVs and emergency visits randomly selected from the emergency visit samples (3659/45,742, 7.99%). Then, we combined the total samples of the unscheduled EDRVs and emergency visits into a single test data set. This study increases in proportion to the sample sizes of unscheduled EDRVs and emergency visits by older adult patients for test data sets by setting the attribute of supervised resample (biasToUniform=200) in the Weka software. After such a resampling procedure, the average number of unscheduled EDRVs and emergency visits by older patients were 7231 and 7153, respectively. We obtained 30 test data sets after 30 repeated resampling and mixed procedures, and the test data sets were used for further decision tree analysis through tenfold cross-validation. In this study, the decision tree achieved an average sensitivity of 76.65% for accurately predicting the unscheduled EDRVs, an average specificity of 76.95% for accurately predicting nonreturn to the ED, and an average overall prediction accuracy of 76.80%.

### Ethics Statement

This study was approved by the institutional review board No. SE20209B of Taichung Veterans General Hospital. As the NHIRD data set comprises deidentified secondary data for research purposes, written consent from the study participants was not obtained, and the institutional review board of Taichung Veterans General Hospital issued a formal written waiver of the need for consent.

## Results

### Decision Tree Analysis

According to the results of gain ratio of the decision tree using C4.5 implemented by Weka J48, the decision tree showed that ES, cancer drug treatment and monitoring, LOSED, cerebrovascular disease, DC, physician year of practice, patient age, LU, x-ray, DS, TC, and hospital level are critical variables for data classification and prediction. The top three influencing variables, in descending order, were ES, chronic illness-cancer drug treatment and monitoring (CICDTM), and LOSED. The 72-hour unscheduled EDRVs by older ED patients was negatively correlated with patients’ ES, positively correlated with their CICDTM, and negatively correlated with their LOSED. This demonstrated that patients from low-income households or those with CICDTM are at a higher risk of unscheduled EDRVs within 72 hours. The likelihood of EDRVs decreased exponentially if older patients had an overnight stay or longer LOSED at their first visit.

### Decision Criteria for Predicting Older Patients’ Unscheduled EDRVs

**Figure 1:**
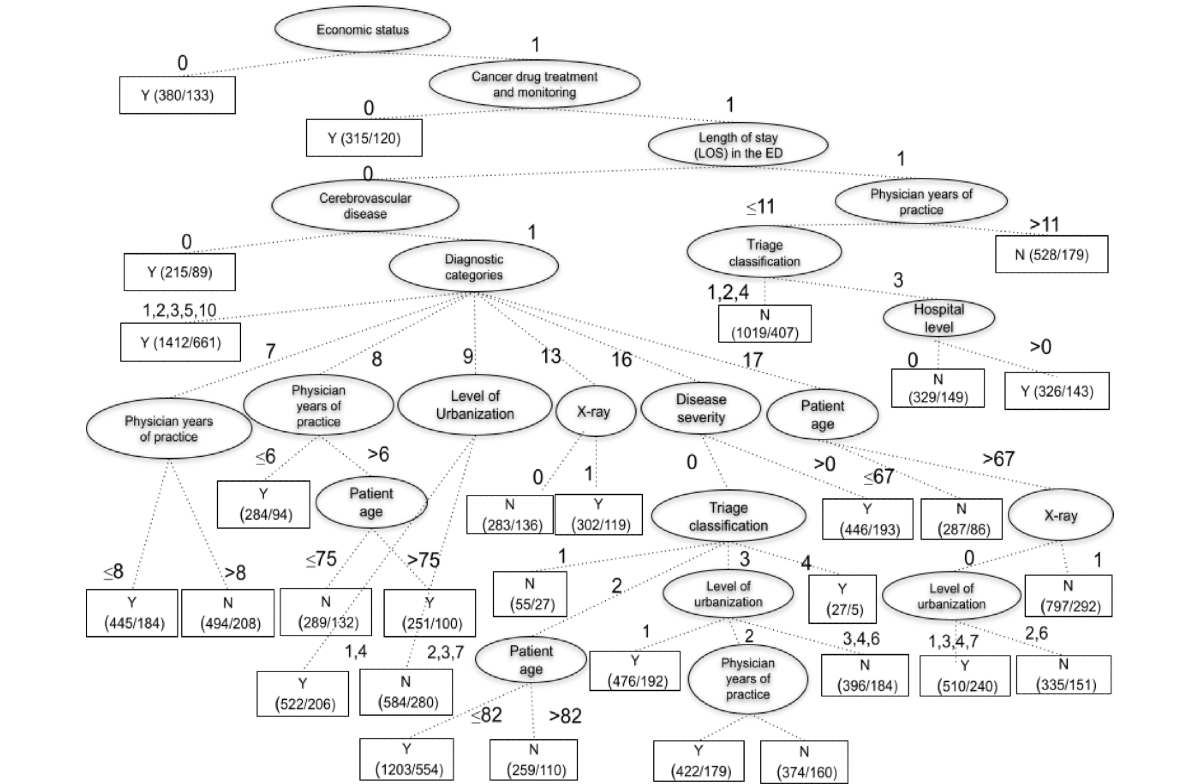
Decision criteria for predicting older patients’ unscheduled emergency department return visits. ED: emergency department; LOS: length of stay.

The decision tree generated 11 prediction patterns (rules) for unscheduled EDRVs in older patients, which are presented in Figure 1.

As shown in the upper section of Figure 1, the top three influencing factors are ES, chronic illness, and LOSED. Each branch of the decision tree represents a decision rule that indicates the decision path of higher possibility or risk within 72-hour unscheduled EDRVs. The front number in the rectangular box represents the EDRV and the other represents the number without EDRV. For example, the left-hand branch of node ES, called Rule 1, represents 380 older adult patients from low-income households with EDRVs and 133 patients without EDRV (Textbox 1). From Rules 3-11, the older patients were not from low-income households and had no cancer drug treatment and monitoring; therefore, these two characteristics are not repeated in the explanation within parentheses.

**Decision criteria for predicting older patients’ unscheduled emergency department return visits.**

**Decision Criteria for Predicting Older Patients’ Unscheduled Emergency Department Return Visits**

- Rule 1: economic status (ES)=0 (older patients from low-income households)
- Rule 2: ES=1 and chronic illness-cancer drug treatment and monitoring (CICDTM)=0 (older patients from non–low-income households with cancer drug treatment and monitoring)
- Rule 3: ES=1, CICDTM=1, length of stay in the emergency department (LOSED)=0, and chronic illness-cerebrovascular disease (CICD)=0 (older patients stay in the emergency department (ED) for less than 1 d, and with cerebrovascular disease)
- Rule 4: ES=1, CICDTM=1, LOSED=0, CICD=1, and diagnostic categories (DC)=1, 2, 3, 5, and 10 (older patients stay in ED less than 1 day, with no cerebrovascular disease but infectious diseases and parasitic diseases, tumor, endocrine and immune diseases, mental illness, and genito-urinary system diseases)
- Rule 5: ES=1, CICDTM=1, LOSED=0, CICD=1, DC=7, and physician year of practice (PYP)≤8 (older patients stay in the ED for less than 1 day, with no cerebrovascular disease but circulatory system diseases, and treated by physician with 8 or fewer years of practice)
- Rule 6: ES=1, CICDTM=1, LOSED=0, CICD=1, DC=8, and (PYP≤6 or PYP>6 and patient age [PA]>75) (older patients stay in the ED less than 1 day, with no cerebrovascular disease but respiratory diseases, and treated by a physician with 6 or less years; or all the conditions are same but treated by a physician with more than 6 years of practice, and PA is more than 75)
- Rule 7: ES=1, CICDTM=1, LOSED=0, CICD=1, DC=9, and level of urbanization (LU)=1, 4 (older patients stay in the ED for less than 1 day, with no cerebrovascular disease but digestive diseases, and live in a high LU or general town)
- Rule 8: ES=1, CICDTM=1, LOSED=0, CICD=1, DC=13, and x-ray=1 (older patients stay in the ED for less than 1 day, with no cerebrovascular disease but musculoskeletal system diseases, had no x-ray)
- Rule 9: ES=1, CICDTM=1, LOSED=0, CICD=1, DC=16, and (disease severity [DS]=0 and triage classification [TC]=3; LU=1 or LU=2 and PYP>8; or TC=2, PA≤82 or TC=4; or DS=1, 2, 3, 4; older patients stay in ED less than 1 day, with no cerebrovascular disease but have signs, symptoms, and diagnosis less clear, and DS, TC of 3, and live in high LU, and treated by physician with more than 8 years of practice and live in Remote town or all conditions are the same with TC=2 and aged 82 or less, or all conditions are the same as TC=4, or all conditions are the same with DS)
- Rule 10: ES=1, CICDTM=1, LOSED=0, CICD=1, DC=17, PA>67, x-ray=0, and LU=1, 3, 4, 7 (older patients stay in the ED for less than 1 day, with no cerebrovascular disease but injury and poisoning, had no x-ray, and lived in a high LU or an emerging town, general town, or remote town)
- Rule 11: ES=1, CICDTM=1, LOSED=1, PYP≤11, TC=3, and hospital level=1, 2 (older patients stay in ED less than 1 day, treated by physician with 11 or fewer years, with TC and visit Regional Hospital or District Hospital)

## Discussion

### Principal Findings

Among the 28 investigated variables, as shown in Multimedia Appendix 1, in patient-related, disease-related, hospital-related, and physician-related characteristics, only 12 variables were identified as critical criteria in the decision tree prediction model. This study found that ES, age, CICDTM, chronic illness-cerebrovascular disease in patient characteristics and TC, DC, x-ray, DS, and LOSED in disease characteristics were the key factors influencing unscheduled EDRVs. In addition, hospital level and LU in hospital characteristics and years of practice in physician characteristics were key predictive factors of unscheduled EDRVs. The results showed that only a portion of the investigated variables in patient-related, disease-related, hospital-related, and physician-related characteristics were key factors influencing older patients’ unscheduled EDRVs. Through the decision tree analysis, this study found 11 useful decision rules for predicting unscheduled EDRVs within 72 hours by the identified factors. The obtained decision rules can be easily applied by physicians and nurses in the ED to evaluate the risk or possibility of unscheduled EDRVs within 72 hours for older patients. ES, CICDTM, and LOSED are highlighted as the top three influencing variables of the prediction model of older patients with unscheduled EDRVs within 72 hours.

In this study, we confirmed that older ED patients with less economic privilege were more likely to return to the ED than those in the opposite group. Furthermore, these findings are consistent with those of a previous study [42]. Possible reasons include that older adults with lower ES have fewer resources to attend to their health and basic preventive health care. They often defer medical treatment and, thus, have a high demand for emergency medical resources. Therefore, the pathological conditions that develop among underprivileged older patients are also more complex than those of their privileged counterparts, which increases the difficulty of treatment and leads to a high rate of EDRVs.

In addition, older patients with chronic symptoms that remain prevalent or frequently relapse may prefer to return to the ED for rapid and convenient treatment, rather than visit an outpatient department. The rates of 72-hour unscheduled EDRVs were higher for older patients who required cancer drug treatment and monitoring or were diagnosed with chronic cerebrovascular diseases. These results confirm the findings of Liaw et al [26], McCusker et al [31], and Wu et al [27]. A possible reason may be that patients with cancer or cerebrovascular disease have a greater need for emergency treatment and hospitalization for pain. In addition, patients diagnosed with chronic cerebrovascular diseases are at a high risk of a second stroke. Active interventions to improve the effectiveness and efficiency of delivering medical education on pain control and stroke prevention can help patients and their family members manage and alleviate this risk and further reduce their EDRVs.

We also found that older patients with shorter LOSED had higher rates of EDRVs than those who stayed in the ED overnight or longer. Patients older than 65 years are known to have a lower metabolic rate [43], and a longer length of stay (LOS) can enable medical staff to conduct more detailed observations of the effectiveness of the provided treatments. It can also further verify the patient’s reaction to the prescribed medicine and modify the types of medicines needed. However, a lack of sufficient ED resources may result in problems such as ED overcrowding, inadequate number of hospital beds, or poor evaluation practices. It may also prevent hospitals from increasing the LOS for older patients in the ED.

In this study, some specific DC (infectious diseases and parasitic diseases, tumors, endocrine and immune diseases, mental illness, circulatory system diseases, respiratory diseases, digestive diseases, genito-urinary system diseases, musculoskeletal system diseases, signs, symptoms and diagnosis less clear, and injury and poisoning) were found to be highly related to unscheduled EDRVs under certain circumstances (patients from non–low-income households and LOS less than 1 day and patients without CICDTM and cerebrovascular disease). The results showed that only a portion of the DC (disease types) [12,25,27] were identified as factors influencing older adult patients’ unscheduled EDRVs; however, some DC were not considered as significant factors in this study.

Older patients classified as class 3 or higher on the TC level had a higher likelihood of 72-hour unscheduled EDRVs if they were treated by physicians with less than 11 years of practice, a result partially consistent with a previous study [15,34]. The conventional emergency medicine curriculum does not include geriatrics; curriculum materials focus on the care of adults aged less than 65 years or children. Meanwhile, the challenges in providing medical services to older patients are highly specific and complex. Physicians who have more years of practice may overcome the problems engendered by the lack of formal geriatric training in emergency medicine, whereas lack of experience in the ED increases the difficulty of accurately diagnosing symptoms in older patients.

Moreover, physicians often underestimate the TC of frail older patients because of the absence of prominent symptoms. This increases the risk of delayed treatment and the likelihood of unscheduled EDRVs. Platts-Mills et al [44] have also asserted that the TC is designed specifically for the general adult population and does not have adequate specificity for the older adult population or reflects the severity of their pathological conditions.

### Limitations

As mentioned above, decision trees have been widely used in various clinical studies, and the analyzed results can be easily applied to clinical practice. Our prediction model developed by the decision tree achieved an acceptable rate for sensitivity, specificity, and overall prediction accuracy. Future researchers can use the results of this study as a reference and apply other methods such as random forest or support vector machine to generate a prediction model and obtain higher accuracy. As data were collected in Taiwan, caution is needed when generalizing the results of this study. Meanwhile, because of the limited content of NHIRD, important variables other than claim-based data cannot be obtained. Furthermore, the insured area and degree of urbanization may be different from the actual area of residence. In addition, the 20,478 records with incomplete or illogical values excluded in this study can cause selection bias. Future studies can use advanced interpolation techniques to explore the characteristics of deleted records and extend the results of this study.

### Conclusions

Compared with previous studies [11,12,14-17], past research samples from a single institution are impossible to discuss patients returning to the ED from different hospitals. This study used the NHIRD to obtain a more comprehensive picture of older adult patients’ EDRVs within 72 hours. This study identified 12 key predicting factors out of the 28 investigated factors and provided 11 decision rules for early detection and possible prevention of unscheduled EDRVs within 72 hours. For example, more attention should be paid to patients aged 65 years and older featured with low-income households or those with CICDTM, and have a reasonably longer LOSED at their first visit.

Medical and health care is an important segment of Taiwan’s *New Southbound Policy* [45] to engage in partnership with 18 countries of the Southeast Asia-Pacific family. The findings of this study can serve as a reference for those countries in the planning and promotion of medical services and age-friendly policies. In countries with rapidly aging populations, the demographics of EDs have shifted substantively toward older patients, and most of their EDs are not fully prepared for the challenge of caring for the aging population. Although the majority of emergency medical curriculum materials focus on the care of children and adults under 65, some countries have begun to integrate geriatrics and emergency medicine training into a defined and validated geriatric emergency medicine curriculum [46]. The Taiwan Society of Emergency Medicine has included geriatrics in its emergency medicine curriculum, with training and emphasis on acute problems in older patients, including medical and intestate ethics and certification of age-friendly health care institutions.

For physicians, our prediction model can be used as a reference to improve medical management and clinical services to reduce older patients’ 72-hour unscheduled EDRVs. Policymakers can use the results of this study to generate incentives for medical institutions to provide appropriate education to older patients and their family members before discharge. Medical institutions may create mechanisms that incorporate our prediction model and develop a decision-making support system for emergency return visits, similar to other clinical decision support systems [47,48]. Such a system can be used in triage procedures for early detection and prevention of unscheduled EDRVs, and it will help health care providers to rapidly identify older patients who are likely to make unscheduled EDRVs and to further reduce the rate of such visits.

In summary, this study is based on large population-based retrospective data from the NHIRD and uses machine learning techniques, which demonstrate superior fault tolerance and learning capability from massive data. The decision tree machine learning technique was further used for data analysis and validation because of its simplicity, interpretability, and applicability of the results compared with other machine learning techniques. Through the decision tree technique, decision rules with important factors influencing the unscheduled EDRV prediction model from the considerations of patient, disease, hospital, and physician characteristics were obtained. The decision rules may serve as a reference for the early detection of unscheduled EDRV in older adults. Further studies can be based on the findings of this study and integrate hospitals’ information systems or electronic medical records to generate appropriate rules for unscheduled EDRVs for older adults in different hospitals.

## Appendix

Multimedia Appendix 1 Patient characteristics and variables.

Multimedia Appendix 2 Vitae.

## Abbreviations

CICDTM: chronic illness-cancer drug treatment and monitoring
DC: diagnostic categories
DS: disease severity
ED: emergency department
EDRV: emergency department return visit
ES: economic status
LOS: length of stay
LOSED: length of stay in the emergency department
LU: level of urbanization
NHIRD: National Health Insurance Research Database
TC: triage classification
