# Supplementary material for: Predicting Unscheduled Emergency Department Return Visits Among Older Adults: Population-Based Retrospective Study
Source: JMIR Med Inform. 2021 Jul 28;9(7):e22491. doi: 10.2196/22491 (PMC8367131; doi:10.2196/22491)
Supplement: Multimedia Appendix 1 [file medinform_v9i7e22491_app1.docx]

**Table 1.** Patient characteristics and variables.

| Characteristics and variable | | | Value (encoding) |
| --- | --- | --- | --- |
| **Patient** | | | |
|  | Gender | | 0 (female)/1 (male) |
|  | Age^a^ | | Patient’s age |
|  | ES^b^ | | 0 (low-income households)/1 (not low-income households) |
|  | Major disease or injury | | 0 (yes)/1 (no) |
|  | **Chronic illness** | | |
|  |  | Hypertension | 0 (yes)/1 (no) |
|  |  | Diabetes | 0 (yes)/1 (no) |
|  |  | Heart disease | 0 (yes)/1 (no) |
|  |  | Bowel dysfunction | 0 (yes)/1 (no) |
|  |  | Cerebrovascular disease | 0 (yes)/1 (no) |
|  |  | Chronic kidney inflammation | 0 (yes)/1 (no) |
|  |  | Vestibular disease | 0 (yes)/1 (no) |
|  |  | Mental illness | 0 (yes)/1 (no) |
|  |  | Arthritis | 0 (yes)/1 (no) |
|  |  | Cancer drug treatment and monitoring | 0 (yes)/1 (no) |
| **Disease** | | | |
|  | TC^c^ | | |
|  |  | | 1=Resuscitation |
|  |  | | 2=Emergent |
|  |  | | 3=Urgent |
|  |  | | 4=Nonurgent |
|  | DC^d^ | | |
|  |  | | 1=Infectious diseases and parasitic diseases (001～139)^e^ |
|  |  | | 2=Tumor (140～239) |
|  |  | | 3=Endocrine and immune diseases (240～279) |
|  |  | | 4=Blood and blood-forming organs diseases (280～289) |
|  |  | | 5=Mental illness (290～319) |
|  |  | | 6=Nervous system diseases (320～389) |
|  |  | | 7=Circulatory system diseases (390～459) |
|  |  | | 8=Respiratory diseases (460～519) |
|  |  | | 9=Digestive diseases (520～579) |
|  |  | | 10=Genito-urinary system diseases (580～629) |
|  |  | | 12=Skin and subcutaneous tissue disorders (680～709) |
|  |  | | 13=Musculoskeletal system diseases (710～739) |
|  |  | | 14=Congenital malformations (740～759) |
|  |  | | 16=Signs, symptoms and diagnosis less clear (780～799) |
|  |  | | 17=Injury and poisoning (800～999) |
|  | **Test items** | | |
|  |  | X-ray | 0 (yes)/1 (no) |
|  |  | Specific angiography | 0 (yes)/1 (no) |
|  |  | Ultrasound | 0 (yes)/1 (no) |
|  | Surgery disposition | | 0 (yes)/1 (no) |
|  | DS^f^ | | |
|  |  | | 0=0 point^g^ |
|  |  | | 1=1 point |
|  |  | | 2=2 points |
|  |  | | 3=3~6 points |
|  |  | | 4=7 points |
|  | **LOS^h^ in the ED^i^** | | |
|  |  | | 0=LOS in the ED less than 1 day |
|  |  | | 1=LOS in the ED more than 1 day |
|  | Medical season | | 1=Spring/2=Summer/3=Fall/4=Winter |
| **Hospital** | | | |
|  | **Hospital level** | | |
|  |  | | 0=Medical Center |
|  |  | | 1=Regional Hospital |
|  |  | | 2=District Hospital |
|  |  | | 3=Primary Health care Unit (Clinics) |
|  | **LU^j^** | | |
|  |  | | 1=High LU |
|  |  | | 2=Middle LU |
|  |  | | 3=Emerging town |
|  |  | | 4=General town |
|  |  | | 5=Aging town |
|  |  | | 6=Agricultural town |
|  |  | | 7=Remote town |
|  | Gender | | 0 (female)/1 (male) |
|  | Years of practice^a^ | | Years of practice in ED |
|  | **Specialty** | | |
|  |  | | 0=Emergency medicine only |
|  |  | | 1=Emergency medicine and other specialties |
|  |  | | 2=Other specialties but no emergency medicine |
|  |  | | 3=No any specialties |
|  | Unscheduled EDRVs^k^ within 72 hours | | 0 (no)/1 (yes) |

^a^Represents numeric variable; others were nominal variables.

^b^ES: economic status.

^c^TC: triage classification.

^d^DC: diagnostic categories.

^e^The value in the parentheses was encoded by International Classification of Diseases,Ninth Revision, Clinical Modification (ICD-9-CM).

^f^DS: disease severity.

^g^The value of DS was calculated by Charlson Comorbidity Index.

^h^LOS: length of stay.

^i^ED: emergency department.

^j^LU: level of urbanization.

^k^EDRVs: ED return visits.
