# Supplementary material for: Predicting Unscheduled Emergency Department Return Visits Among Older Adults: Population-Based Retrospective Study
Source: JMIR Med Inform. 2021 Jul 28;9(7):e22491. doi: 10.2196/22491 (PMC8367131; doi:10.2196/22491)
Supplement: Multimedia Appendix 2 [file medinform_v9i7e22491_app2.docx]

### Vitae

**Rai-Fu Chen** is an associate professor in the Department of Information Management at Chia Nan University of Pharmacy and Science, Taiwan. He received a PhD in Information Management from National Chung Cheng University, Taiwan, in 2008. His research interests include decision support systems, health care information systems, and electronic medical records. His research has appeared in the International Journal of Medical Informatics, BMC Medical Informatics and Decision Making, JMIR Medical Informatics, Telemedicine and eHealth, among others.

**Kuei-Chen Cheng** is a PhD student in the Department of Information Management at National Chung Cheng University. Her research interests include health management and long-term care.

**Yu-Yin Lin** received her MS degree in Information Management from the National Chung Cheng University, Taiwan, in 2013. Her research interests include medical informatics, data mining, and information retrieval.

**I-Chiu Chang** is a professor of MIS at the National Chung Cheng University, Taiwan. She received her PhD from the University of Texas, Arlington, USA. Her research interests include decision support systems, health care information systems, and electronic medical records. Her published works have appeared in Decision Support Systems, Information & Management, and Government Information Quarterly.

**Cheng-Han Tsai** is a PhD student at the Department of Information Management at National Chung Cheng University and a chief physician of the Department of Emergency in Chiayi Branch, Taichung Veterans General Hospital, Taiwan. His research interests include administrative data analysis and medical informatics.
